# Comparison of Trifecta Outcomes in Standard Versus Mini Percutaneous Nephrolithotomy for Renal Stone Management

**DOI:** 10.7759/cureus.80328

**Published:** 2025-03-10

**Authors:** Immad Ahmed, Liaqat Ali, Abdul Haseeb, Khizer Zaman, Nauman Ul Mulk, Fayyaz Ullah, Muhammad Raheel, Muhammad Zohaib, Jamal A Shah

**Affiliations:** 1 Department of Urology, Hayatabad Medical Complex, Peshawar, PAK; 2 Department of Urology, Leicester General Hospital, University Hospitals of Leicester (UHL) NHS Trust, Leicester, GBR

**Keywords:** endoscopic stone surgery, endourology, percutaneous nephrolithotomy (pcnl), trifecta, urolithiasis

## Abstract

Introduction: Urolithiasis is a common urological condition, and percutaneous nephrolithotomy (PCNL) is a widely used treatment option for renal stones. The trifecta analysis, which includes the complete stone-free rate (SFR), absence of complications (Clavien-Dindo classification), and no need for auxiliary procedures, provides a standardized method for comparing outcomes. This study evaluates the trifecta outcomes of standard PCNL versus mini PCNL

Methodology: This prospective cohort study was conducted at the Department of Urology, Institute of Kidney Diseases, Peshawar, from January 2022 to March 2024. A total of 180 consecutive patients who underwent PCNL were enrolled in the study. Using a lottery method, patients were randomly assigned to two equal groups (standard PCNL and mini PCNL). Both groups consisted of 90 patients each. Patients who required a change in the planned surgical procedure were replaced with new participants from the sample frame. A structured proforma was used to record preoperative, perioperative, and postoperative data for the trifecta analysis. Data were analyzed using IBM SPSS Statistics for Windows, Version 23.0 (Released 2015; IBM Corp., Armonk, NY, United States). Logistic regression was performed to assess predictive factors for the trifecta, and the odds ratio (OR), confidence interval (CI), and p-value were calculated.

Results: The mean age in the standard PCNL group was 43.21 ± 3.51 years vs 44.03 ± 3.17 years in the mini PCNL group (p = 0.10). The mean stone size in the standard PCNL group was 30.62 ± 5.88 mm vs 30.28 ± 6.03 mm in the mini PCNL group (p = 0.70). The mean stone density in the standard PCNL group was 1366.25 ± 74.28 HU vs 1342.66 ± 107.34 HU in the mini PCNL group (p = 0.08). Stones were completely cleared in 84 (93.3%) patients in the standard PCNL group and 69 (76.7%) in the mini PCNL group (p = 0.02). Auxiliary procedures, including extracorporeal shock wave lithotripsy (ESWL), ureteroscopy (URS), and repeat double J (DJ) stenting, were required in four patients (4.4%) in the standard PCNL group compared to 20 patients (20%) in the mini PCNL group. Regarding complications, the standard PCNL group recorded complications in 16 patients (17.77%), including Grade 1 (six patients), Grade 2 (five patients), and Grade 3 (five patients), according to the Clavein-Dindo classification. In the mini PCNL group, six patients (6.66%) experienced postoperative complications, including Grade 1 (five patients) and Grade 2 (one patient) (p = 0.02). The overall trifecta success rate was 71.12% in the standard PCNL group vs 50.03% in the mini PCNL group.

Conclusion: The trifecta analysis indicates that standard PCNL has a higher SFR, while mini PCNL is safer but requires more auxiliary procedures.

## Introduction

Globally, kidney stone formation is a prevalent urological condition, accounting for 80%-90% of all urinary calculi [1]. According to guidelines, percutaneous nephrolithotomy (PCNL) is the recommended treatment for renal stones larger than 20 mm. Standard PCNL (24-30 Fr) remains the preferred treatment for large renal stones due to its superior stone-free rates (SFRs) [2-4].

Mini PCNL was first introduced by Jackman et al. as an alternative to standard PCNL [5]. The European Association of Urology (EAU) defines mini PCNL as a procedure using a tract size smaller than 22 Fr [6]. The mini PCNL procedure has shown a better complication profile compared to the standard PCNL. However, concerns have been raised about its effectiveness in stone clearance, particularly for large-sized calculi, as the smaller tract may limit the maneuverability of instruments required for their removal [7].

Indeed, one significant obstacle in resolving this controversy lies in the heterogeneity of outcome metrics used for PCNL [8]. To address this, the authors proposed a composite outcome measure, the PCNL trifecta, which includes complete stone-free status, no complications (as per the Clavien-Dindo classification), and no need for auxiliary procedures (e.g., extracorporeal shock wave lithotripsy (ESWL), flexible ureteroscopy (URS), or repeat double J (DJ) stenting) together [9]. Despite existing comparisons between standard and mini PCNLs, the lack of standardized reporting and inconsistent outcomes has limited the ability to draw definitive conclusions [10].

This study aims to conduct a comparative trifecta analysis of standard PCNL and mini PCNL to evaluate their effectiveness in renal stone management.

## Materials and methods

Study design and setting

This study was executed as a single-institution prospective cohort study at the Department of Urology, Institute of Kidney Diseases, Peshawar, Pakistan, between January 2022 and March 2024. It focused on patients eligible for PCNL as a treatment for stone stones. After obtaining IREB approval from the Institute of Kidney Diseases (Certificate No. 341), all participants were provided a detailed explanation of the study's objectives, methodology, potential benefits, and risks before giving their informed consent.

Sample size calculation and patient selection

The sample size was determined using the OpenEpi calculator (Dean AG, Sullivan KM, Soe MM. OpenEpi: Open Source Epidemiologic Statistics for Public Health, Version. www.OpenEpi.com, updated 2013/04/06). A total of 180 consecutive patients scheduled for PCNL were selected from an initial sample frame of 200. To maintain random allocation, a simple random sampling technique using the lottery method was applied. Patients were equally divided into two groups: the standard PCNL group and the mini PCNL group, each consisting of 90 patients.

Patients who required a change in the planned surgical procedure were excluded and replaced with new participants from the sample pool.

Inclusion and exclusion criteria

Patients aged 16-65 years with renal stones larger than 20 mm in diameter or a density greater than 1,000 HU were included. Exclusion criteria included patients with residual stones following prior PCNL or other endourological procedures, active untreated urinary tract infections or sepsis, or deranged coagulation profiles.

Surgical procedure

To minimize variability in outcomes, all surgical procedures were standardized and performed under general anesthesia by three experienced consultant urologists. Two urologists performed both standard and mini PCNLs, while one specialized in standard PCNL exclusively. All three urologists had more than seven years of experience in endourology. Under general anesthesia, the patient was placed in the lithotomy position. A cystoscopy was performed, and a 6-Fr ureteric catheter was inserted over a guidewire into the renal pelvicalyceal unit under fluoroscopic control. A per urethral Foley catheter was placed for drainage, and the patient was then turned prone for percutaneous access.

Percutaneous access was achieved using an 18-G needle under fluoroscopic guidance. Contrast was then administered via the ureteric catheter to visualize the pelvicalyceal system. Once correct access was confirmed, a 0.035-inch glide wire was introduced into the collecting system or ureter. The needle was withdrawn, and an incision was made through the skin and fascia. Tract dilation was performed according to the surgical technique: for mini PCNL, dilation was carried out using Cook fascial dilators and a single-step dilator up to 16-20 Fr, while for standard PCNL, the tract was dilated to 30 Fr using Alken dilators. An appropriately sized Amplatz sheath was then inserted to maintain access.

Stone fragmentation and retrieval were performed using different instruments depending on the technique. In mini PCNL, a 12-Fr nephroscope was used, while in standard PCNL, a 24-Fr nephroscope was utilized for larger stone fragments. Stone fragmentation was achieved using a pneumatic lithoclast, and fragments were retrieved with forceps or by a retrograde saline push through the ureteric catheter.

All patients underwent antegrade placement of a 6-Fr DJ stent and nephrostomy tube insertion to ensure proper urinary drainage and minimize complications. Patients were closely monitored for any immediate postoperative issues, with discharge planned once they were medically stable, following the removal of the catheter and nephrostomy tube. All patients were scheduled for follow-up after one month, during which stone clearance was assessed using a repeat non-contrast CT scan.

Stone-free status was defined as the complete absence of residual calculi or the presence of clinically insignificant residual fragments (CIRFs), which were asymptomatic, nonobstructive, and less than 4 mm in size. Any intraoperative or postoperative complications occurring within 30 days were documented and classified according to the Clavien-Dindo classification. Patients with significant residual stones were offered additional therapies, such as ESWL or repeat DJ stenting.

Statistical analysis

Data analysis was performed using IBM SPSS Statistics for Windows, Version 23.0 (Released 2015; IBM Corp., Armonk, NY, United States). Categorical variables were compared using chi-square tests, and independent t-tests were used for parametric continuous variables. A p < 0.05 was considered statistically significant, ensuring that the observed differences between groups were not due to random variation. Logistic regression was used to assess predictive factors influencing trifecta outcomes, with odds ratios (ORs), confidence intervals (CIs), and p-values reported.

## Results

This study compared two groups of patients undergoing different PCNL techniques, focusing on stone characteristics, SFRs, the need for additional procedures, postoperative complications, and overall trifecta success. Both groups had similar baseline characteristics. The mean stone size was 30.62 ± 5.88 mm in the standard PCNL group and 30.28 ± 6.03 mm in the mini PCNL group, with no significant difference (p = 0.70). The mean patient age was 43.21 ± 3.51 years in the standard PCNL group and 44.03 ± 3.17 years in the mini PCNL group (p= 0.10), showing no statistically significant difference. Stone density was also comparable between the two groups (p = 0.08). However, Guy’s Score distribution varied significantly (p = 0.01), indicating potential differences in stone complexity between the groups.

Stone clearance and auxiliary procedures

Stone-free status was achieved in 93.3% (84/90) of patients in the standard PCNL group compared to 76.7% (69/90) in the mini PCNL group (p = 0.02). This suggests that standard PCNL was more effective in clearing stones. The need for auxiliary procedures (such as ESWL, URS, or repeat DJ stenting) was also significantly lower in the standard PCNL group. Only four patients (4.4%) required additional interventions compared to 20 patients (20%) in the mini PCNL group.

Postoperative complications

Complications were categorized using the Clavien-Dindo classification. The standard PCNL group had a slightly higher incidence of minor complications (Clavien-Dindo Grade 1), occurring in six patients (6.7%), compared to five patients (5.6%) in the mini PCNL group. Moderate complications (Clavien-Dindo Grade 2) were observed in five patients (5.6%) in the standard PCNL group, whereas only one patient (1.1%) in the mini PCNL group experienced similar issues. Severe complications (Clavien-Dindo Grade 3), requiring surgical or radiological intervention, occurred in five patients (5.6%) in the standard PCNL group, but none was reported in the mini PCNL group. This difference in overall complication rates was statistically significant (p = 0.03). The interventions required for the five patients in the standard PCNL group were as follows: (1) one patient developed postoperative ipsilateral pleural effusion due to an upper pole puncture, which was managed with a chest tube placed under local anesthesia. (2) Two patients developed postoperative abdominal distention due to irrigating fluid extravasation, managed with an open abdominal drain placed under local anesthesia uneventfully. (3) Two patients had postoperative perinephric collection/abscess, which was treated with an ultrasound-guided percutaneous drainage tube placed under local anesthesia uneventfully. All patients recovered without further issues.

Trifecta analysis

When evaluating trifecta outcomes (complete SFR, no complications, and no need for additional procedures), the standard PCNL group had a significantly higher success rate (71.12%) compared to the mini PCNL group (50.03%) (p = 0.01). To calculate the trifecta failure rate, we summed the rates of individual unachieved parameters: incomplete stone clearance (7.7%), redo procedures (4.4%), and all complications (17%), resulting in a total failure rate of 29%. Consequently, the trifecta success rate (cases achieving all parameters) in the standard PCNL group was 71% and 50.03% in the mini PCNL group. Logistic regression analysis identified significant predictors of trifecta success. Standard PCNL was associated with higher odds of achieving trifecta success compared to mini PCNL (OR = 2.5, 95% CI 1.3-4.7, p = 0.01). Lower Guy’s Score and smaller stone size were also predictive of trifecta success (p = 0.01). These results suggest that standard PCNL not only achieved better stone clearance but also minimized the need for further interventions, leading to more favorable overall surgical outcomes. All the details are given in Table 1.

**Table 1:** Patient characteristics and intraoperative and postoperative parameters PCNL: percutaneous nephrolithotomy.

Parameter	Standard PCNL (n = 90)	Mini PCNL (n = 90)	p-value	Statistical test
Age (years)	43.21 ± 3.51	44.03 ± 3.17	0.10	Independent t-test
Stone size (mm)	30.62 ± 5.88	30.28 ± 6.03	0.70	Independent t-test
Stone density (HU)	1366.25 ± 74.28	1342.66 ± 107.34	0.08	Independent t-test
Guy's Score			0.01	Chi-square
Grade 1	0 (0%)	33 (36.7%)		
Grade 2	4 (4.4%)	57 (63.3%)		
Grade 3	68 (75.6%)	0 (0%)		
Grade 4	18 (20.0%)	0 (0%)		
Stone clearance	84 (93.3%)	69 (76.7%)	0.02	Chi-square
Auxiliary procedures	4 (4.4%)	18 (20.0%)	0.01	Chi-square
Complications (Clavien-Dindo)			0.02	Fisher's exact
Grade 1	6 (6.7%)	5 (5.6%)		
Grade 2	5 (5.6%)	1 (1.1%)		
Grade 3	5 (5.6%)	0 (0%)		
Trifecta success rate	63 (71.12%)	45 (50.03%)	0.01	Chi-square

## Discussion

Renal stone disease is a global health concern, affecting approximately 5% of the US population, with staghorn calculi comprising 10%-20% of cases. These stones require effective management to prevent serious complications [11]. PCNL techniques, including standard PCNL, mini PCNL, and micro PCNL, each have distinct indications and outcomes [12]. While SFR is widely used as a primary surgical outcome, there is no universally standardized definition. Therefore, trifecta analysis, which evaluates stone clearance, complication rates (Clavien-Dindo classification), and the need for auxiliary procedures, has been proposed as a more comprehensive measure of PCNL success [13,14].

In our study, standard PCNL achieved a significantly higher SFR (93.3%) compared to mini PCNL (76.6%) (p = 0.02), supporting the notion that standard PCNL leads to superior stone clearance. Similar findings were reported by ElSheemy et al., who also observed higher SFRs with standard PCNL compared to mini PCNL [7]. Additionally, Wishahi et al. reported an 80% SFR for mini PCNL and 85% for standard PCNL [15]. However, our results contrast with those of Abdelhafez et al., who found a 74.6% SFR for mini PCNL and a lower rate of 61.3% for standard PCNL [16]. These discrepancies highlight the importance of patient selection and surgical expertise in determining outcomes.

We also observed higher complication rates in the standard PCNL group compared to the mini PCNL group, based on the Clavien-Dindo classification. The overall complication rate was 17.7% in the standard PCNL vs 6.66% in the mini PCNL group (p = 0.03). Most complications in the standard PCNL group were Clavien-Dindo Grades 2 and 3, whereas the mini PCNL group mainly experienced Grade 1 complications, reinforcing the idea that mini PCNL is a safer procedure. Similar trends were observed by Mazzon et al., who found lower complication rates in mini PCNL compared to standard PCNL [17]. Additionally, Qin et al. noted higher rates of hemoglobin drop and blood transfusion requirements in standard PCNL compared to mini PCNL [10]. The need for secondary procedures was significantly lower in the standard PCNL group (p = 0.01). In the mini PCNL group, patients often required auxiliary procedures such as ESWL and URS to achieve complete stone clearance. Khadgi et al. similarly found that the need for secondary procedures was higher in mini PCNL compared to standard PCNL, particularly in cases of staghorn stones [18].

The overall trifecta success rate, defined by complete stone clearance, no complications, and no need for additional procedures, was significantly higher in the standard PCNL group (71.12%) compared to the mini PCNL group (50.03%) (p = 0.01). These results emphasize that while mini PCNL is a safer, less invasive approach, standard PCNL remains superior in terms of stone clearance and reducing the need for auxiliary procedures.

Our findings underscore the importance of standardizing outcome criteria in PCNL research. The choice between standard PCNL and mini PCNL should be guided by factors such as stone size, density, and complexity. Standard PCNL remains the preferred option for large and dense stones due to higher SFRs, despite higher complication risks. The mini PCNL, on the other hand, is a less invasive technique with fewer complications, making it a suitable choice for smaller stones [6]. These insights can assist endourologists in tailoring treatment strategies to optimize both stone clearance and patient safety in renal stone management.

Limitations

Our study has several important limitations. First, as it was conducted at a single center in Peshawar, the findings may not fully represent broader populations or different healthcare settings, thus limiting the generalizability of the results. Future multicenter studies with larger, more diverse patient populations are needed to enhance the generalizability of these findings.

Second, we used a pneumatic lithoclast for stone fragmentation in both standard and mini PCNLs. Although the pneumatic lithoclast is widely used, emerging technologies such as the holmium:YAG laser may offer improved outcomes. Furthermore, the lithotripters with suction capabilities may provide a significant advantage during mini PCNL. Exploring different fragmentation methods could provide valuable insights into their impact on stone clearance, complications, and overall surgical outcomes.

Finally, the procedures in our study were performed by multiple surgeons, which naturally introduced some variation in technique and decision-making. Surgeon experience and individual preferences could have influenced clinical outcomes. Future studies with a standardized surgical approach or stratified analysis based on surgeon expertise would help provide more consistent and reliable conclusions.

## Conclusions

Our study comparing the trifecta outcomes of standard PCNL and mini PCNL found that standard PCNL offers superior stone clearance and requires fewer auxiliary procedures to achieve higher SFRs. In contrast, while mini PCNL is a safer procedure with fewer complications, it often necessitates multiple auxiliary interventions to achieve optimal stone clearance.
